# How a distractor influences fixations during the exploration of natural scenes

**DOI:** 10.16910/jemr.10.2.2

**Published:** 2017-04-10

**Authors:** Hélène Devillez, Anne Guérin-Dugué, Nathalie Guyader

**Affiliations:** University of Colorado Boulder, USA; Université de Grenoble-Alpes, France

**Keywords:** Natural scenes, Free exploration, Eye Movement, Distractor Effect, Fixation control, Saliency, Statistical modeling

## Abstract

The distractor effect is a well-established means of studying different aspects of fixation programming during the exploration of visual scenes. In this study, we present a taskirrelevant distractor to participants during the free exploration of natural scenes. We investigate the control and programming of fixations by analyzing fixation durations and locations, and the link between the two. We also propose a simple mixture model evaluated using the Expectation-Maximization algorithm to test the distractor effect on fixation locations, including fixations which did not land on the distractor. The model allows us to quantify the influence of a visual distractor on fixation location relative to scene saliency for all fixations, at distractor onset and during all subsequent exploration. The distractor effect is not just limited to the current fixation, it continues to influence fixations during subsequent exploration. An abrupt change in the stimulus not only increases the duration of the current fixation, it also influences the location of the fixation which occurs immediately afterwards and to some extent, in function of the length of the change, the duration and location of any subsequent fixations. Overall, results from the eye movement analysis and the statistical model suggest that fixation durations and locations are both controlled by direct and indirect mechanisms.

## Introduction

Human vision during visual perception is an active process where observers select information relevant to their exploration goal ( 5 ). When viewing a visual scene, we typically make three to four eye fixations per second. At each fixation, decisions are made regarding the next fixation, and the next saccade is programmed. This programming involves the decision to terminate the current fixation (when) and the choice of location for the next fixation (where). Both characteristics have been widely studied, most of the time separately, in order to gain better understanding of saccade programming during scene exploration ( 17, 30 ). The scene onset delay paradigm has shown that fixation durations can be divided into two populations: one population which comes under the direct control of the scene and which increases in duration as the delay is increased, and a second population, not under the direct control of the current scene, whose duration does not increase, irrespective of scene presence. These results support a mixed eye movement control model ( 7, 8 ). Other paradigms have been proposed to study which factor influences saccade programming and fixation duration. The *remote distractor effect* is a well-known phenomenon where saccadic responses to a visual target are delayed if a distractor and the target are flashed simultaneously. Results have clearly shown that remote distractors modified not only saccade trajectory and landing location ( 25, 11 ), but also saccade latency ( 29, 28, 16 ). It has also been shown that the time needed to select the next fixation and to program the next saccade was impacted by the distance of the distractor from the locations of the central fixation and the saccade target ( 15 ). Saccade amplitudes can also be also impacted, as saccades tend to land at intermediate locations between the saccade target and the distractor location ( 29 ).

The effect of a distractor has also been studied using more ecological paradigms during which observers explored natural scenes. This effect is called the *distractor effect*. Authors using these paradigms deal with fixation durations rather than saccade latencies because there is no explicit saccade target. Brockmole and Henderson ( 1 ) found that an object, which appeared after 500 ms of exploration, captured the attention immediately, and suggested that transient motion captures attention in a bottom-up manner.

In a series of experiments, Pannasch and colleagues used digitized paintings and a gaze contingent irrelevant distractor onset ( 20, 19, 18 ). They showed that any visual change prolongs the current fixation duration in comparison to previous and subsequent fixations. This result suggests that fixation durations are under the direct control of stimulus information ( 19 ). The distractor effect on fixation duration was analyzed in relation to a number of different factors. The authors tested different stimulus onset asynchronies and showed similar distractor effects for each of these asynchronies. They also measured the influence of the distractor on fixation durations in relation to the amplitude of the preceding saccade, and showed that visual distractors had significantly more influence if the amplitude of the previous saccade was less than 5° ( 20 ). In addition, they found that the effect of the distractor on fixation duration was stronger when it was close to the current fixation ( 18 ).

However, while the effect of a distractor on the duration of a current fixation has been widely studied, the influence of the distractor on subsequent fixations has been relatively neglected. The question of whether distractor onset, known to increase the duration of the current fixation, also influence the programming of the next saccade has yet to be investigated. It has been shown that a distractor flashed during a fixation increases the duration of the fixation. We also know that the next fixation is programmed during the current fixation. We therefore wondered if the distractor location would become a potential target location for the next fixation. In our data analysis, we explored the link between the increase in the duration of the current fixation and the programming of the next saccade, leading to the next fixation location, in function of distractor duration. We asked if a distractor that strongly increases fixation duration had a greater chance of being gazed at in subsequent fixations, and for how long a distractor needed to be displayed in order to be targeted during subsequent fixations. In this study, we aimed to investigate the control and programming of successive fixations during the exploration of natural scenes through the appearance of an irrelevant distractor. A Gabor patch was flashed at the onset of a fixation which occurred at the beginning of exploration. Three different durations were used for the distractor: the distractor appeared and disappeared within the fixation; the distractor was present during the whole fixation; or the distractor never disappeared. These three conditions were compared to a control condition during which no distractor was flashed.

We began by studying the distractor effect on classical eye movement parameters (fixation durations and fixation locations). This allowed us to validate the proposed protocol by replicating the distractor effect during the free exploration of natural scenes. We measured how the distractor modified the fixations that followed its onset, and the link between the distractor effect on the current fixation duration and the location of subsequent fixations. We hypothesized that if the distractor had an effect on the current fixation, measured by an increase in duration, it would be gazed at more often during subsequent fixations. We already know that the next fixation location is chosen during the current fixation. We might suppose that the increase in duration of the current fixation is due to the fact that the distractor attracts visual attention, and therefore becomes a potential target location for the next fixation. We tested our hypothesis using several distractor durations. One could legitimately hypothesize that when a distractor is presented for a longer duration, the visual system has more time to encode its precise location. If this was the case, we would observe a greater impact on fixation locations which followed, with more fixations landing on the distractor.

Secondly, we proposed a simple statistical model to evaluate the contribution of a distractor to fixation locations observed, relative to scene saliency. It is already well known that fixations are driven, at least in part, by the saliency of the scene, which is defined by the locations that attract observers’ gaze ( 14, 23, 13 ). The model also allowed us to evaluate the distractor effect on fixations other than those that landed on the distractor location. The proposed model assumes that recorded fixations might be explained by a linear combination of two guiding factors represented by 2D spatial maps: (1) the region of interest of the scene evaluated by experiment saliency maps and (2) the influence of the distractor evaluated by a localized Gaussian function. The influence of each factor was evaluated using the Expectation-Maximization (EM) algorithm. This algorithm has been successfully applied in visual attention models ( 27, 9, 2, 6, 3 ).

## Material and methods

### Stimuli

**Figure 1:**
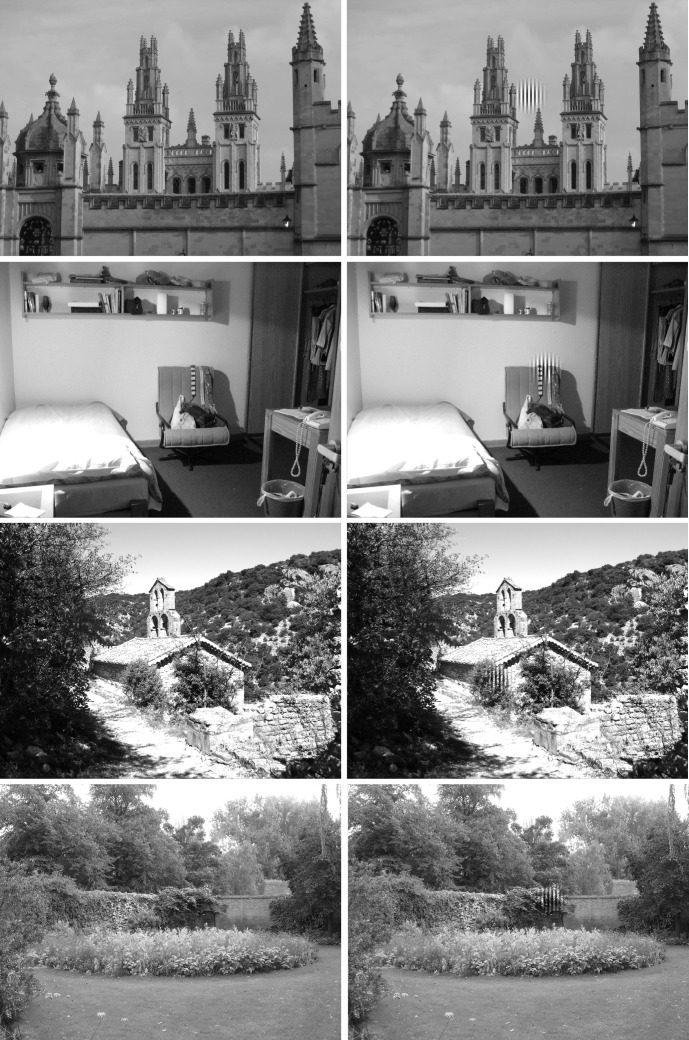
Examples of scenes without (left column) and with the distractor (right column); distractors always appeared 4° from the scene center.

We used 156 real-world images representing a large variety of scenes (landscapes, buildings, indoor scenes) (Figure 1). Scenes were presented in grayscale and had a resolution of 768 by 1024 pixels and were presented in full screen subtending a visual angle of 30° × 40°. All scenes were equalized to an average luminance of 127 (luminance values were between 0 and 255).

### Distractor

The distractor, a Gabor patch, was inserted into the scene. The patch had a radius R_d_ of 2.2° (i.e. 56.4 pixels) with maximal luminance contrast. The distractor shape corresponded to a 2D Gaussian function modulated by a vertical sinusoid with a spatial frequency of 2.2 cycles per degree. It appeared at the onset of the second detected fixation^1^. Note that “fixation 1” refers to the first fixation that occurred after scene onset (and not the fixation that started before scene onset). To speed up the display of the scene with the distractor at the second fixation, this scene was computed before the experiment. For each scene, four different locations for the distractor were chosen randomly on a 4° radius-centered circle.

### Apparatus

Eye movements were recorded using the SR Research Eyelink II (500 Hz) infrared eye tracking system. Stimuli were presented on a 20-inch ViewSonic CRT monitor, with a resolution of 768 by 1024 pixels, a refresh rate of 85 Hz, at a viewing distance of 57 cm. Experiments were run using SoftEye ( 10 ).

### Protocol

Each trial started with a white fixation cross presented for 1 s on a gray screen. This fixation cross was located on the screen diagonals 5° from the center. After 1 s, and if gaze had stabilized for 100 ms (gaze contingent display), the scene was displayed. If the participant did not gaze at the cross, the scene was still displayed after 5 s, but the trial was considered invalid and recorded data were not analyzed.

**Figure 2:**
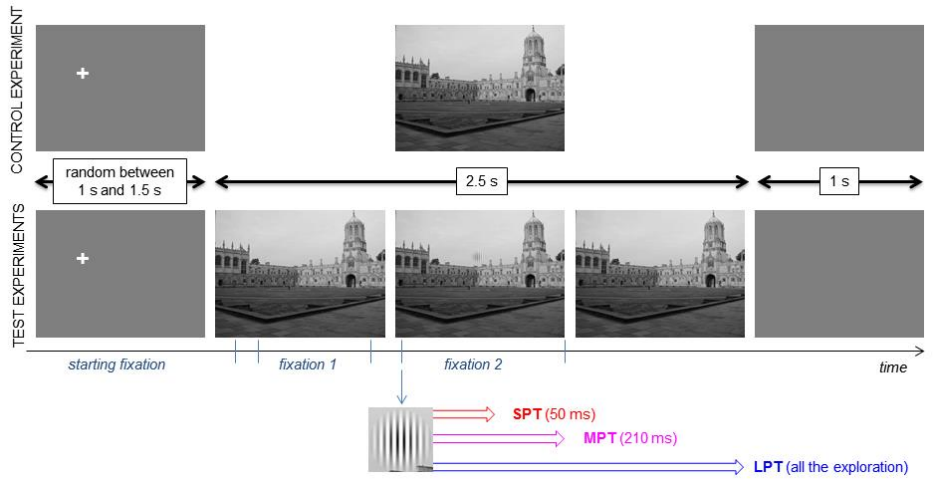
Trial sequence with distractor onset triggered on fixation 2. In the control condition, scenes were displayed without the distractor for 2.5 sec. In distractor conditions, the distractor was displayed for three different durations: 50 ms (Short Presentation Time), 210 ms (Medium Presentation Time) and during the whole exploration (Long Presentation Time).

In the control condition, the scene was displayed for 2.5 s. Three distractor conditions were used. The distractor always appeared at the onset of fixation 2, an early fixation known to be mainly guided by the visual properties of the scene. As already mentioned, the starting point of exploration was on the screen diagonals 5° from the center of the screen. The probability that fixation 1 would appear on the scene center was therefore very high, due to the “central bias” observed during eye movement experiments ( 24 ). The distractor was displayed for 50 ms (Short Presentation Time: SPT), 210 ms (Medium Presentation Time: MPT) or until the end of the exploration (Long Presentation Time: LPT). The duration of scene presentation was therefore different in these three distractor conditions. In the SPT condition, scenes containing a distractor were displayed for 50 ms, and then, scenes with no distractor were shown for 2250 ms. In the MPT condition, a scene featuring the distractor was displayed for 210 ms and followed by the same scene without the distractor for 2090 ms. In the LPT condition, a scene with the distractor was displayed for 2300 ms. Distractor onset was synchronized with the onset of fixation 2 for each scene and each observer. On average, it appeared 580 ms after scene onset. Finally, at the end of the trial, a gray screen appeared for 1 s (Figure 2).

Observers were asked to look carefully at scenes in preparation for a questionnaire on scene content. The questionnaire was never actually given. A 9-point calibration routine was completed and repeated every 50 trials or if the drift correction, completed every ten trials, detected an error above 0.5°.

### Participants

Forty-eight naïve healthy volunteers with normal or corrected-to-normal vision took part in the experiment (17 female; age range: 21-27, M=24.12, SD=2.46). Participants were assigned randomly to one of four experimental conditions: the control condition (without the distractor) or one of the three distractor conditions (SPT, MPT or LPT). In the control condition, participants viewed all 156 scenes. In the distractor conditions, each participant viewed only 52 of the 156 scenes. In the MPT and LPT conditions, all participants reported perceiving the distractor. However, in the SPT condition only 10 out of 12 reported it. We analyzed only the data from these 10 participants in order to maintain consistency. Analyses indicated that the two observers who did not notice the distractor differed from the other participants in their eye movement patterns but showed no increase in fixation durations.

### Data analysis

Raw eye-movement data were preprocessed by removing fixations that occurred around eye blinks or outside the presentation screen. Fixations whose duration was shorter than 50 ms or longer than 1000 ms were excluded. In total, less than 10% of the data was removed, including invalid trials.

Because the distributions of fixation durations were skewed, median values were used for each subject per condition. Eye fixation locations were also extracted and the proportion of fixations that landed on the distractor was computed. In order to test if the proportions of fixations that landed on the distractor location were greater in the distractor conditions than in the control condition, we calculated the proportion of fixations recorded at the distractor location in the control condition (with no distractor). Fixations were represented by a circle of 1° around the fixation location and classified as being on the distractor if there was an intersection between the circle with a radius of 2.2° representing the distractor and the circle with a radius of 1° representing the fixation.

For each scene, we computed a map highlighting all the regions of interest. These maps were called empirical saliency maps. They were created using fixations recorded during the control condition. Empirical saliency maps were obtained for each scene by summing a 2D Gaussian with a standard deviation of 1° centered on fixations. The size of Gaussians was chosen in relation to foveal size and eye-tracker accuracy. It should be noted that only fixations 2 to 8 were used; we did not use fixation 1, mainly due to central bias ( 24 ). Empirical saliency maps were then converted into binary maps using a threshold of 0.2 (for eye movement analysis) or normalized to 1 to correspond to probability density function (for the modeling part). These maps obtained using the eye fixations of several observers predict the fixations of other observers about as well as the best saliency models do. Even if individual differences do exist between observers, consistency between the fixations of several observers has been reported, making the prediction of fixation locations of one observer by using the fixations of other observers efficient ( 26, 12 ).

## Results: Eye movements

The distractor effect was measured on both fixation durations and locations based on the fixation order in scene exploration. Analysis of the distractor effect also took into account the saliency of the scene, computed by empirical saliency maps.

Analyses were conducted using a repeated measures Analysis of Variance (ANOVA).

### Fixation duration

**Figure 3:**
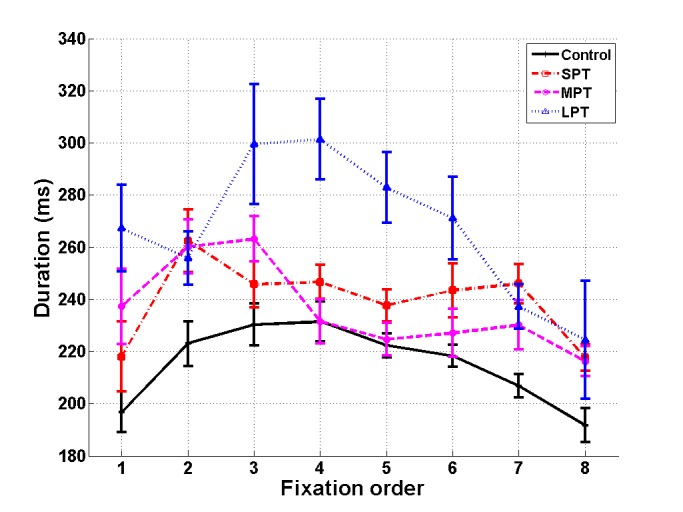
Mean fixation duration in milliseconds as a function of fixation order (fixations 1 to 8) for the control condition and the three distractor conditions (SPT, MPT and LPT). Error bars represent standard error.

A repeated-measure ANOVA was conducted with Fixation (fixation 1 to 8) as a within-subjects factor and Condition (control/SPT/MPT/LPT) as a between-subjects factor. We found main effects of *Condition*, F(3,41) = 9.65, p < .001, η_p_² = 0.41, and Fixation, F(7,287) = 10.59, p < .001, η_p_² = 0.20. The interaction Condition × Fixation was also significant, F(21,287) = 2.47, p < .001, η_p_² = 0.15 (Figure 3). Multiple comparisons of distractor conditions to the control condition were assessed with Dunnett post-hoc tests. The distractor effect was particularly noticeable at fixation 2, during which the distractor appeared. Its duration significantly increased in the three distractor conditions compared to the control condition (p < .05 – marginal effect for LPT, p = 0.06). We observed an equal increase of the duration of fixation 2 in the three distractor conditions. The fixation following distractor onset (fixation 3) also increased in duration compared to the control condition in the MPT and LPT conditions (p < .05). Moreover, in the LPT condition, fixations 4 to 8 were longer than in the control condition (p < .05). Finally, the fixation preceding distractor onset (fixation 1) was more prolonged in the LPT condition than in the control condition (p < .05).

### Fixation location

**Figure 4:**
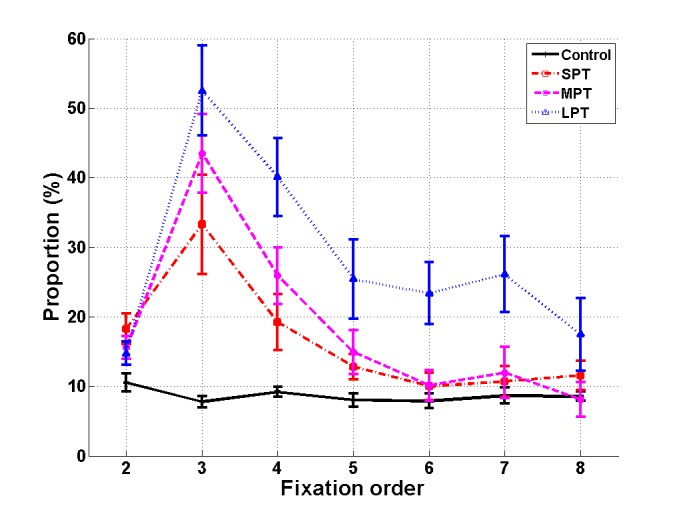
Mean proportion of fixations on the distractor location as a function of fixation order (fixations 2 to 8) for the control condition and the three distractor conditions (SPT, MPT and LPT). Error bars represent standard error.

A repeated-measure ANOVA was conducted with Fixation (fixation 2 to 8) as a within-subjects factor and Condition (control/SPT/MPT/LPT) as a between-subjects factor. The analysis revealed main effects of Condition, F(3,41) = 9.55, p < .001, η_p_² = 0.41, and Fixation, F(6,246) = 37.82, p < .001, η_p_² = 0.48. The interaction Condition × Fixation was also significant, F(18,246) = 6.78, p < .001, η_p_² = 0.33 (Figure 4). Multiple comparisons of distractor conditions to the control condition were assessed with Dunnett post-hoc tests. The proportion of fixation 3s on the distractor, i.e. the fixation immediately following distractor onset, was higher in all distractor conditions compared to the control condition (p < .01). The proportion of fixation 4s on distractor location was also higher for MPT and LPT than in the control condition (p < .01). In the LPT condition, the proportion of fixations 5 to 7 that landed on the distractor was again higher than in the control condition (p < .05).

Results on fixation durations and fixation locations were coherent: the current fixation increased in duration and the following fixation had a high probability of landing on the distractor location. The following analysis directly tested the link between the distractor effect on the duration of fixation 2 and the location of fixation 3.

#### Relation between fixation 2 duration and fixation 3 location

Three separated chi-square tests were conducted to test the independence of fixation 2 duration (Short/Long) and fixation 3 location (In/Out). Fixation 2 duration was divided into two groups according to the duration of fixation 2 during the Control condition (Short: < 223 ms; Long: > 223 ms). There was a dependence link between fixation 2 duration and fixation 3 location for MPT, χ^2^ = 9.44, p < 0.01, and LPT, χ^2^ = 34.49, p < 0.001. For these conditions, trials with long fixation 2s also showed more fixation 3s inside the distractor location. No dependency link between fixation 2 duration and fixation 3 location was observed for SPT, χ^2^ = 0.29, p = 0.86 ns.

#### Fixation 3 location and saliency

A repeated-measure ANOVA was conducted on fixation 3 location, with *Saliency* (Salient/Non salient distractor) as a within-subjects factor and *Condition* (SPT/MPT/LPT) as a between-subjects factor. Statistical analysis revealed a main effect of *Saliency*, F(1,31) = 22.53, p < .001, η_p_² = 0.42. No effect of *Condition*, F(2,31) = 1.95, p = .16 ns, η_p_² = 0.11, was observed and the interaction *Saliency × Condition* was not significant, F(2,31) = .47, p = .63 ns, η_p_² = 0.03. Multiple comparisons were assessed with Bonferroni post-hoc tests. For MPT and LPT, the proportion of fixation 3s on the distractor was higher when the distractor location was salient (p < .05).

### Interim summary

In line with previous studies ( 12, 13 ), an increase in the mean duration of fixation 2s and a high probability of observing fixation 3s on the distractor location, were observed. These parameters were linked when the distractor was presented for longer durations: more fixation 3s were observed inside the distractor location when fixation 2s were of longer duration. Although the effect of the distractor was the same on the duration of fixation 2 in the three distractor conditions, we observed differences on subsequent fixations. Distractors of longer duration had a greater impact on the location and even on the duration of subsequent fixations. Finally, the distractor effect was influenced by scene saliency at the distractor location: a stronger distractor effect was observed with an increase in the proportion of fixation 3s on the distractor location, when the distractor appeared in a salient location. We measured the distractor effect only on fixations that landed on the distractor location with these eye movement analyses. Furthermore, the analysis presented did not allow us to quantify the contribution of the distractor in relation to scene saliency, which is known to drive fixation location during the exploration of scenes.

## Statistical modeling

In this section, we proposed a mixture model to quantify the relative importance of scene saliency and the presence of the distractor on fixation locations. This model focused only on fixation locations and did not take into account fixation durations. One important property of the model was its capacity to capture the influence of the distractor on all fixations, including fixations which did not land on the distractor location. Furthermore, the model evaluated the effect of the distractor at onset and on subsequent fixations.

### The proposed model

The proposed statistical model explains the distribution of recorded eye fixation locations using a linear weighted summation of three possible guiding factors. We used the Expectation-Maximization (EM) algorithm, a statistical method using the recorded eye movements to calculate the relative contribution of each guiding factor in order to maximize the global likelihood of the mixture model ( 21 ).

The first factor known to guide eye movement during scene exploration is the saliency of a scene. We used the experiment saliency map: S_m_(p), with p=(x,y) the 2D fixation position.

The second factor in the model was the impact of distractor^2^ appearance on a given position. In line with previous results showing the attractiveness of the distractor, this factor was modeled by a 2D Gaussian function: Ν(p;μ(t),σ(t)). In other words, μ(t) (mean) was the spatial position and the parameter σ(t) (standard deviation) represented the size of the Gaussian. The parameters μ(t) and σ(t) were estimated for each fixation order . This factor acted as a “localizer Gaussian” and indicated that fixation locations were gathered on a particular spatial location modeled by the Gaussian. The ability of this factor to reflect the influence of the distractor on fixation locations was related to the parameters μ(t), σ(t) of the Gaussian function. For this reason we named this second factor the “Gaussian” and not the “distractor” factor.

Finally, even if the saliency of the scene and the distractor were the two main factors which explained eye movements, a third factor was added to cover any other non-controlled processes which may have influenced eye movements. This third factor was a noise factor, and explains any fixations that were not explained by the two other factors. The lower the weight of this factor was, the better the other factors explained the data. It was simply modeled by a uniform spatial distribution on the whole image: U(p) and was called “noise factor” for simplicity.

The guiding factors that explain fixation locations after distractor onset could be confounded: when the distractor appeared in a salient location, the contributions of the scene saliency and the distractor could not be distinguished. Consequently, only trials where the distractor was not in a salient location were used in the proposed model. 49% of the trials were therefore used. This also allowed us to avoid instability in the convergence of the parameters of the model.

The density function f(p,t) of the fixation position p=(x,y) at each fixation order t was expressed in terms of an additive mixture of the three spatial maps, each associated with a given prior probability or weight α_i_(t),i = 1,2,3:

f(p,t)=α₁(t)×S_m_(p)+ α₁(t)×Ν(p;μ(t),σ(t))+α₃(t)×U(p)

with p=(x,y) the 2D fixation position, f(p,t) the eye fixation map for one distractor condition (SPT, MPT, or LPT) at each fixation order t (t= 2:8) and α_i_(t) the estimated weights of the factor with α₁(t)+α₁(t)+α₃(t) = 1. α₁(t) represented the weight of the saliency map, α₁(t) the weight of the Gaussian factor and α₃(t) the weight of the “noise factor”.

The EM algorithm was used for each distractor condition, for each fixation order, to calculate unknown parameters. To deal with the sensitivity of the estimated parameters to initial conditions, ten randomly chosen values for the initialization were used.

The parameters μ(t) and σ(t) of the Gaussian factor, which were free parameters across fixation order, were calculated. For each fixation order , and each distractor condition, the number of estimated parameters was six (α₁(t),α₁(t),α₃(t),μ_x_(t),μ_y_(t) and σ(t) with σ_x_(t)=σ_y_(t)=σ(t)), and five degrees of freedom (α₁(t)+α₁(t)+α₃(t)=1). The attractiveness of the distractor was analyzed by both the weight, and the spatial parameters (μ(t),σ(t)) at the convergence. Consequently, interpretation of this factor as a guiding factor during the exploration could be carried out only once estimated parameters had been obtained at the EM convergence. More precisely, the interpretation of this “localized Gaussian” factor as the “distractor factor” was only justified when the mean corresponded to the physical position of the distractor. To evaluate it, the Euclidean distance d_μ_(t)=d_E_(μ(t),D₀) between μ(t) and the spatial location D₀ of the distractor, was computed, in function of the fixation order t. If this distance was different from zero, the nature of this factor changed. In other words, its interpretation depends on the distance d_μ_(t). If the distance is high, this Gaussian factor does not represent the distractor accurately, but rather indicates that fixation locations are gathered at a particular spatial location modeled by the Gaussian function. That is why we chose to call this guiding factor the “localized Gaussian” factor.

### Results

**Figure 5:**
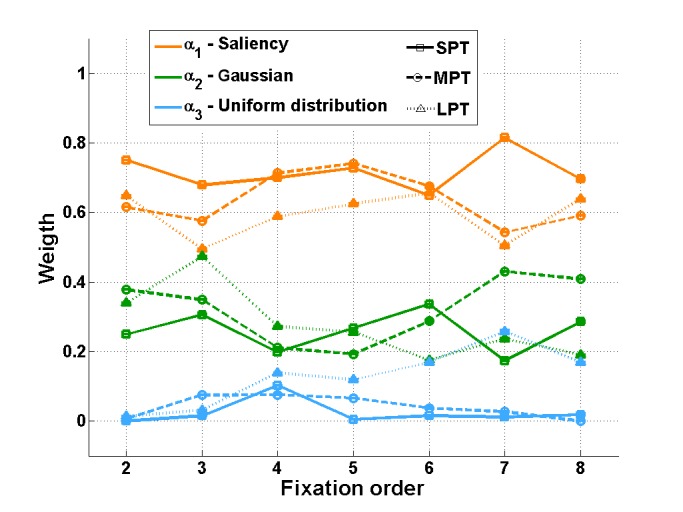
Contributions (weights) of the three factors for the three distractor conditions SPT, MPT and LPT as a function of fixation order. Mean estimations across initializations and corresponding standard errors are plotted. Note that standard error bars are really small.

**Figure 7:**
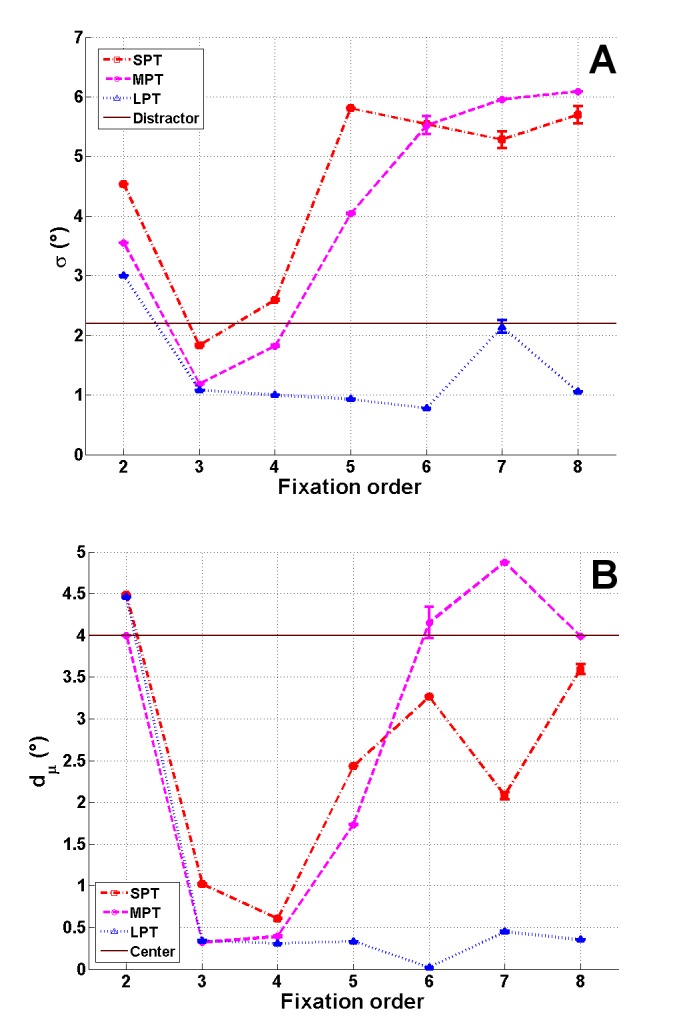
Evolution of σ(t) (**A**) and d_μ_(t) (**B**), in degrees, for the three distractor conditions SPT, MPT and LPT as a function of fixation order. Mean estimations across initialization and corresponding standard errors are plotted. The dark blue line shows the radius R_d_=2.2° of the distractor (**A**) and the distance 𝐷₀ of the distractor from the image center (**B**).

The saliency map weight α₁(t) was the highest for all fixations and distractor conditions (Figure 5). Although the scene saliency map had the highest weight, the weight of the Gaussian factor was not negligible. As expected, the temporal evolution of the Gaussian factor (σ(t) and d_μ_(t) was different in the various distractor conditions (Figure 7).

**Figure 6:**
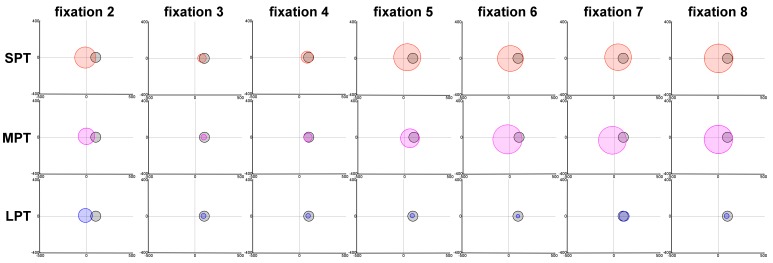
Spatial representation of the evolution of the Gaussian factor, for the three distractor conditions SPT, MPT and LPT as a function of fixation order. The colored circles show the estimated Gaussian factor and are located at the spatial position (μ_x_(t),μ_y_(t)) and with a size σ(t); these parameters were evaluated by the model.. The black circle shows the distractor location with it size R_d_.

In the three distractor conditions, at fixation 2, the standard deviation σ(2) was larger and the distance d_μ_(2) close to zero, showing a mode close to the image center (Figure 6). At this fixation, the distractor had not yet been displayed. The fact that this mode was close to the scene center illustrated the central bias currently observed in eye movement data ( 24 ).

For SPT and MPT conditions, the evolution of σ(t) and d_μ_(t) was divided into three phases. Firstly, between fixations 2 and 3, the Gaussian weight α₁(t) increased slightly (Figure 5). At the same time, the deviation σ(t) decreased and became smaller than the size R_d_ of the distractor. Secondly, after fixation 3, d_μ_(t) decreased to almost zero and σ(t) was slightly larger than R_d_ (Figure 7). In the MPT condition, the Gaussian factor was more attractive, as we can see for fixations 3 and 4. We observed smaller values for σ(3) and σ(4), which in turn were even smaller than R_d_ (Figure 7A). The mean μ was at the location of the distractor (Figure 6). From fixations 4 to 6, σ(t) and d_μ_(t) increased, resulting in the Gaussian factor moving towards the image center (Figure 6). Finally, from fixation 6, all parameters were stabilized.

For the LPT condition, the Gaussian factor weight α₁(t increased from fixation 2 to fixation 3, until it was almost equal to the weight α₁(3) of the saliency mode. The contribution of saliency remained stable, while from fixation 3, the contribution of the distractor decreased, and the “noise factor” weight α₃ increased (Figure 5). From fixation 3, we also observed a decrease in the parameter σ(t) and a decrease in the distance d_μ_(t) (Figure 7). σ(t) which was twice as small as R_d_ from fixation 4 to the end of the exploration. Contributions of this factor and of the “noise factor” were similar from fixation 6. Furthermore, the position of the Gaussian mode converged at the spatial location D₀ of the distractor (Figure 6).

Interestingly, when comparing the three distractor conditions, we observed that the larger distractor effect on eye movements observed in LPT than in MPT and in MPT than in SPT was illustrated by the results of the model. The influence of the distractor on fixation 3, α₁(3), was higher in LPT than in MPT, which in its turn was slightly higher than in SPT.

## General Discussion

The present study demonstrates that presenting a taskirrelevant distractor during the exploration of natural scenes prolongs not only the duration of the current fixation but also modifies both the duration and location of subsequent fixations. After a brief summary of the results, we discuss our findings relative to the programming of eye movements during scene viewing.

Firstly, we replicated previous results which showed an increase in current fixation duration due to distractor onset ( 19 ). More interestingly, we analyzed the effect of the distractor on the duration and location of subsequent fixations, using three durations of distractor. We reported different effects of the distractor depending on its duration. For SPT, only the current fixation duration was impacted, while for MPT, the fixation directly following distractor onset was also lengthened even if the distractor was no longer present. The duration of all fixations increased in the LPT condition compared to the control condition, with larger increases for the two fixations directly following distractor onset.

Secondly, analysis of fixation locations following distractor onset revealed that the distractor spatially attracted fixations. As observed for fixation durations, the attractiveness of the distractor was again stronger when the distractor was presented for a longer duration. Furthermore, we observed that fixations had a greater tendency to land on the distractor location when this location was salient. In order to evaluate the influence of the distractor while taking into account scene saliency, we used a mixture model and the Expectation-Maximization algorithm to calculate the relative importance of these two factors (attractiveness of the distractor, and of salient regions) for eye fixations, when the distractor was not located in a salient region. If we had not done so, it would have been difficult to distinguish the effects of the two factors. Results first showed that fixation locations in the experiment were mainly driven by the saliency of the scene ( 21 ). This was an expected result which supported the validation of our model. In addition to the analysis of fixation locations, the modeling results also showed a strong attractiveness of the distractor (even when it was no longer present) not only for the fixation directly following its onset but also for the one after that. Therefore, even if fixations did not land directly on the distractor location, the results from the statistical model showed that they were still attracted by it for at least two fixations. Distractor attraction was also dependent on duration of presentation: it was stronger in MPT than in SPT. The LPT can be seen here as a control condition, since the distractor remained present for the whole exploration. In this case, the irrelevant distractor attracted fixations for all fixations after its onset.

Several studies have suggested that fixation duration can come under direct or indirect control mechanisms ( 22 ). Direct control theories suppose that decisions about fixations are made during the current fixation and so individual fixations are controlled by visual and cognitive factors associated with the scene region currently under fixation. On the contrary, indirect control theories suppose that the current fixation is influenced by other factors. In our study, we tested whether the duration and location of a fixation also reflected the processing demands of the previous fixation i.e. whether fixation 3s and fixations which followed were impacted by the distractor which appeared during fixation 2s. If mechanisms that control fixations were direct, the onset of the distractor would not have modified the following fixations. If, on the contrary, mechanisms were indirect, the fixation following distractor onset could potentially be influenced by the distractor and its perceptual trace. Interestingly, distractor onset increased the current fixation duration equally, irrespective of the duration of distractor presentation. These results confirmed that it was the transient motion signal itself which captured attention in a bottomup manner. However, the duration of an irrelevant distractor differentially impacted subsequent exploration. Surprisingly, when the distractor was presented for a longer duration (in the MPT condition), it also impacted the duration of subsequent fixations. Even though it had already disappeared, the distractor remained as a perceptual trace. This was true only when the distractor was flashed for a certain duration, suggesting that when flashed for a short period of time, its onset and the transient motion associated impacted only the current fixation duration. For a longer duration, we might suppose that more information was processed during the current fixation. This information was retained and indirectly increased the next fixation duration. We also observed that a greater number of fixations inside the distractor location when the duration of the previous fixation had been more greatly increased by the distractor. The programming of fixation locations was linked to events which occurred previously in the exploration, and any abrupt and timelimited visual change such as distractor onset modified the subsequent exploration of the scene. Overall, these results suggest that fixation durations and fixation locations are controlled by both direct and indirect mechanisms. Direct control on the current fixation was reflected by the increase in duration and by the location of the next fixation, strongly attracted by the distractor. Indirect control was shown by the residual influence of the distractor on both fixation duration and fixation location, for several fixations after distractor onset and offset.

Our data also support the theory of parallel programming of saccades during scene viewing. The increase in the duration of the current fixation can be seen as a delay in the programming of the subsequent saccade, due to processes involved in cancelling the ongoing saccade and programming the new saccade toward the distractor. These results are also consistent with the assumption of the CRISP model which shows that saccade programming is completed in two stages: an initial, labile stage that is subject to cancellation and a subsequent, nonlabile stage ( 17 ). In our experiment, the distractor probably appeared during the labile stage, at the beginning of a fixation. This is supported by results showing that this subsequent fixation was mainly attracted by distractor location and happened to land exactly in the location of the distractor, in a large number of trials.

Our results could have important implications for understanding how fixations are controlled during the exploration of a complex visual scene. While substantial research has been devoted to investigating the temporal control of fixations separately from the spatial control of fixations, our present study provides evidence on the spatial and temporal aspects of fixation control during the exploration of natural scenes. Furthermore, a statistical model such as the one proposed in the study has several advantages: it is parsimonious, easy to use, and the interpretation of results is straightforward. It uses simple and unrestricted numbers of hypotheses concerning the visual factors that could influence fixation, and quantifies these contributions for all fixations involved in the exploration. Different factors might be included in such a simple model. For example, central bias could be included as a factor or a face saliency map could be added as a possible guiding factor to explain eye fixations. Another possibility is the inclusion of maps depicting specific regions of interest, for example, to quantify the relative importance of a specific object compared to scene saliency. Our study provides a complementary approach to the classical analysis of fixation locations as it allows for the study of all fixations, even those which did not land on the distractor in our particular case. As such, it has the potential to be useful in visual attention research to help gain better understanding of the control and programming of fixations during scene viewing.

## Acknowledgements

The authors would like to thank Sebastian Pannasch for his enlightening comments on the manuscript. This work was supported by a grant from the *French Ministère de la Recherche et de l’Enseignement Supérieur* funding the doctorate work of Hélène Devillez. The software development was performed by Gelu Ionescu.

## Footnotes

There was a delay of 33 ms between fixation onset and the appearance of the distractor.

Due to the protocol, the distractor appeared at a random location, 4° from the center of the screen. Because in the model, the distractor was modeled by a unique spatial mode, we performed a rotation of the data to place the distractor on the right side of the center, still at 4°. Thus, the distractor was always at the same reference position for all trials and all scenes. We analyzed the corresponding fixation locations in relation to this location.
